# Sagittal Posture Parameters of the Spine and Exposure to Awkward Postures in Mattress Manufacture Workers: An Analytical Cross-Sectional Study

**DOI:** 10.3390/jfmk11010087

**Published:** 2026-02-20

**Authors:** Sergio Hijazo-Larrosa, María Orosia Lucha-López, Sofía Monti-Ballano, Eva Barrio-Ollero, César Hidalgo-García, Begoña Martínez-Jarreta, Lucía Vicente-Pina, José Miguel Tricás-Moreno

**Affiliations:** 1 Unidad de Investigación en Fisioterapia, Departamento de Fisiatría y Enfermería, Universidad de Zaragoza, Domingo Miral, 50009 Zaragoza, Spain; shijazo@unizar.es (S.H.-L.); hidalgo@unizar.es (C.H.-G.); l.vicente@unizar.es (L.V.-P.); jmtricas@unizar.es (J.M.T.-M.); 2 Spin off Centro Clínico OMT-E Fisioterapia SLP, Universidad de Zaragoza, Domingo Miral, 50009 Zaragoza, Spain; 3 Departamento de Anatomía e Histología Humanas, Universidad de Zaragoza, Domingo Miral, 50009 Zaragoza, Spain; evbarrio@unizar.es; 4 Grupo de Investigación Consolidado GIIS-063, Instituto de Investigación Sanitaria de Aragón, Avda. San Juan Bosco, 13, 50009 Zaragoza, Spain; mjarreta@unizar.es

**Keywords:** awkward postures, sagittal posture, occupational health

## Abstract

**Background:** Awkward postures are among the most prevalent ergonomic risk factors in occupational environments, including industrial settings. Conventional ergonomic risk assessments rarely address the relationship between sustained awkward postures and alterations in sagittal spinal curvatures. The primary objective of this study was to analyze the association between sagittal spinal posture parameters and exposure to awkward postures in male workers from the mattress manufacturing industry. The potential confounding effects of age, job seniority, body mass index (BMI), and physical activity level were also examined. **Methods:** An analytical cross-sectional study was conducted. Data collected included age, job seniority, anthropometric variables, and physical activity level. Sagittal spinal posture parameters—head alignment, thoracic kyphotic curvature, lumbar lordotic curvature, and pelvic tilt—were assessed using photogrammetry. Exposure to awkward postures was recorded according to occupational health surveillance criteria. **Results:** A total of 116 male workers were randomly selected. BMI showed a significant negative association with head alignment (*p* = 0.001), with a medium effect size (η^2^ = 0.090). Lower BMI values (β = −0.517) were observed in association with a more posterior head position. In addition, participants not exposed to awkward postures presented, on average, a 6.479° lower thoracic kyphotic curvature angle compared with exposed workers (*p* = 0.050), indicating a greater kyphotic curvature among those exposed. **Conclusions:** In this sample, lower BMI was associated with a more posterior head position and improved alignment with the upper trunk. Furthermore, exposure to awkward postures was related to a modest increase in thoracic kyphotic curvature, suggesting postural adaptations to occupational demands.

## 1. Introduction

The term occupational health frames the relationship between work and health. It is defined [1] as the field of knowledge in which different entities converge in order to promote, protect, and restore the health of the population in relation to work. The concept of health surveillance also encompasses activities that stem from occupational health and aim to identify health problems and evaluate preventive interventions [2]. The Occupational Safety and Health Administration (OSHA) has developed 12 key intermediate action items aimed at minimizing or eliminating safety and health risks [3]. In the field of occupational risk prevention, the occupational risk factor differs from the risk itself in that the former represents the principle or potential cause of harm, while the risk is the probability of that harm occurring [4].

The controlled and structured list of terms (Thesaurus) of the European Agency for Safety and Health at Work (EU-OSHA) defines occupational risk as a dangerous situation, either in the short or long term, and includes chemical, biological, psychosocial, and physical hazards [5]. This has been the conventional classification of risk factors, although in recent years there has been talk of interventions framed within the relationship between work-related stress and epigenetic plasticity factors, with a holistic perspective, through the concept of “Total Worker Health” (TWH) [6].

Awkward postures can be defined as the result of the physical demands imposed on the person performing the tasks and that person’s ability to adopt specific techniques to perform the assigned tasks [7]. Awkward postures are understood to be fixed or restricted body positions, postures that overload muscles and tendons, postures that load joints asymmetrically, and postures that produce static load on the muscles [8]. They are also known as painful positions [9] and are considered one of the biomechanical stress factors that can cause musculoskeletal disorders (MSDs). MSDs are defined by the National Institute for Occupational Safety and Health (NIOSH) as a group of symptoms and injuries that affect the musculoskeletal system and associated structures [10,11]. In the European Union, workers experience this stress with a score of 0.27 (scale of 0 to 1), with little variation between countries or regions [9]. Awkward postures constitute a significant issue in numerous occupational scenarios. They are especially common in manufacturing [12], and static or sedentary works seem to have a greater incidence among them [13]. According to the sixth edition of the European Working Conditions Survey from 2015, 43% of European workers are exposed to the risk of awkward postures for at least a quarter of their working day [13].

The National Safety Council (NSC) considers that awkward postures can result in postural alterations [14]. These can cause alterations in the spine. [15]. In addition, some studies of spinal injuries include awkward postures as an influencing factor [16]. For example, low back pain [17], lumbar hernias with radiculopathy [18], cervical degenerative myelopathy [19], cervical spondylosis [20], cervical hernias [21], and degeneration of the cervical and lumbar intervertebral discs [22,23,24].

Recognizing the broad impact of awkward postures in the spine, this study’s primary objective is to determine the relationship between the sagittal posture parameters of the spine and the exposure to awkward postures in mattress manufacture male workers. We will also analyse the influence of age, job seniority, body mass index (BMI) and the intensity of physical activity as potential confounding variables.

## 2. Materials and Methods

### 2.1. Design and Participants

We designed an analytical cross-sectional study to investigate the relationship between the sagittal posture parameters of the spine and the exposure to awkward postures in mattress manufacture male workers. This study also considered sociodemographic characteristics such as age, job seniority, body mass index and the intensity of physical activity as potential confounders. Before starting the study, it was approved by the Local Ethics Committee “Comité Ética de Investigación Clínica de Aragón (CEICA)” (Protocol Code: PI18/363/ date of approval: 16 January 2019). Written informed consent was obtained from all subjects participating in the study.

To calculate sample size, the expected proportion used was the proportion of the population that may be at elevated risk of trunk-related musculoskeletal injury, associated with various occupational postures (40%) [25]. Assuming a desired precision of +/−10 units percent units, a confidence level of 0.95, and a substitution rate of 20%, the required sample size was estimated at 116 participants. The calculation was performed using the GRANMO calculator (https://www.datarus.eu/en/applications/granmo/) (accessed on 7 March 2019).

From the mattress manufacturing company, a total of 116 male workers were selected through stratified random sampling, ensuring representation from all job categories within the company. This selection represented a subset of the total population of 406 individuals engaged in manufacturing, maintenance, and storage processes within the company.

Inclusion criteria required participants to be between 18 and 61 years old and not currently on sick leave due to illness or work-related injury. Exclusion criteria included a history of spinal surgery, the presence of red flags related to spine issues, or an inability to complete the evaluation and/or questionnaires.

### 2.2. Measures and Data Collection

Data were collected through individual interviews where participants reported their age and job seniority.

Moreover, body weight and height were measured in subjects wearing light clothing and no shoes. Data were collected according to the protocol of the International Society for the Advancement of Kinanthropometry [26]. Height was measured with a wall stadiometer (Seca-707: removable stadiometer)(Seca, Hamburg, Germany), scope 30–220 cm, and weight with an electronic scale (Biological Company: TANITA TBF 300) (Tanita Corporation, Tokyo, Japan). BMI was calculated as weight in kilograms divided by the square of the height in meters.

The assessment of physical activity was conducted using the long version of the International Physical Activity Questionnaire (IPAQ) [27]. The updated Spanish-adapted version, dated October 2002, was employed (https://sites.google.com/view/ipaq/download?authuser=0) (accessed on 10 March 2019). This long version encompasses five distinct domains of physical activity, each evaluated independently with reference to activities performed during the preceding week. The measurement unit applied in this scale is the Metabolic Equivalent of Task (MET). The long form was deemed the most suitable instrument for capturing detailed information across the various dimensions of physical activity [27]. For the purposes of this study, the total intensity of physical activity was calculated and expressed as MET-minutes per week.

The sagittal-plane postural parameters of the spine were evaluated using photogrammetry. Reflective markers (B&L Engineering^®^. Pinsco, Inc. dba B&L Engineering, Santa Ana, CA, USA) were placed on the following anatomical landmarks:

Left earlobe;

Spinous process of the seventh cervical vertebra (C7);

Spinous process of the second thoracic vertebra (T2);

Spinous process of the ninth thoracic vertebra (T9);

Spinous process of the twelfth thoracic vertebra (T12);

Spinous process of the first sacral vertebra (S1);

Left anterior superior iliac spine (ASIS);

Left posterior superior iliac spine (PSIS).

The C7 spinous process, the earlobe, the ASIS, and the PSIS were identified following the recommendations of Krawicky et al. [28]. The spinous processes of T2, T9, T12, and S1 were identified according to the methodology proposed by Ferreira et al. [29].

The markers consisted of a 19.05 mm flexible base with a 19-mm sphere, and were affixed to the skin at the specified anatomical points using double-sided adhesive tape.

**Figure 1:**
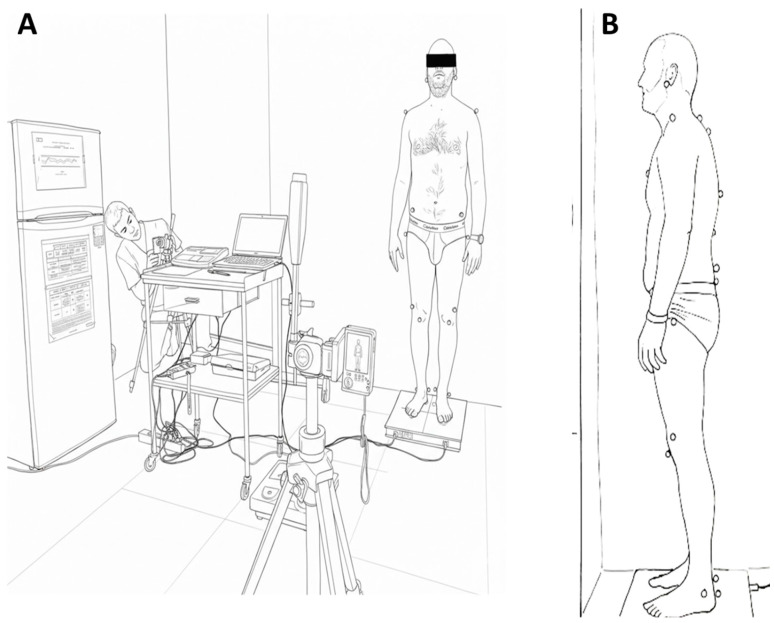
(**A**) Laboratory setup. (**B**) Marker system.

The photographic protocol required participants to remain in a static upright position [30], standing on the foot marks of the Satel^®^ stabilometric platform (40 Hz C3) (SATEL SARL; Blagnac, France), which positioned the feet with 2 cm between the heels and 30° of external rotation. In this position, participants remained still for 5 [31] to 10 [32] seconds while the photograph was taken. The camera was mounted on a tripod at a fixed height of 1 m [33] and placed at a horizontal distance of 2 m from the subject, aligned with the sagittal plane on the left side [28] (Figure 1).

The photographs were analyzed using AutoCAD^®^ software(AutoCAD 2019, version 23.0) (Autodesk, Inc., San Francisco, CA, USA) or the Postural Assessment Software (PAS/SAPO) (version 1.0.x) (https://bmclab.pesquisa.ufabc.edu.br/sapo-en/) (accessed on 20 April 2020), developed by researchers at the University of São Paulo, Brazil [34]. This freely available software allows for the measurement of body distances and angles from digital images for postural evaluation [29]. It is widely used in clinical practice and research and is accompanied by scientific tutorials [29].

**Figure 2:**
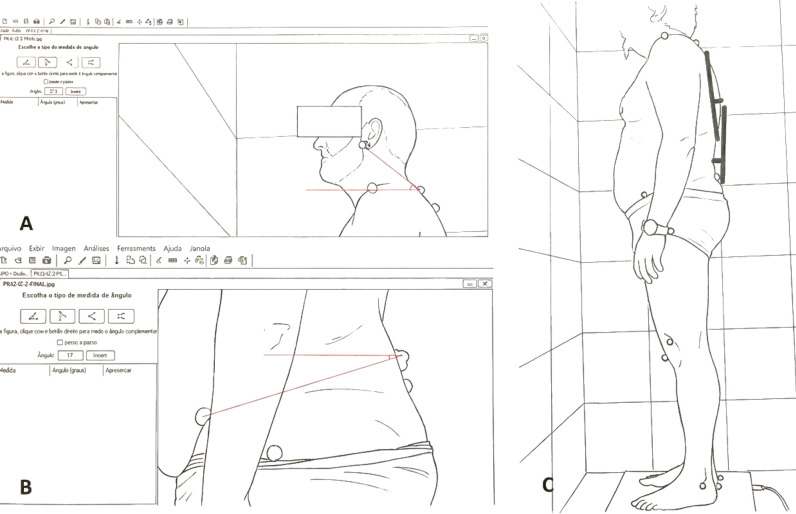
Measurement of the parameters. (**A**) Horizontal head alignment. (**B**) Horizontal pelvic alignment. (**C**) Thoracic kyphotic and lumbar lordotic curvatures.

Using the photographs and markers, the following sagittal-plane parameters were quantified [28,35] (Figure 2):

• Horizontal head alignment: Defined as the angle formed between the line connecting the earlobe and the spinous process of C7 and a horizontal reference line. Lower angles indicate a more forward head position relative to the upper trunk, reflecting anterior head posture. Higher angles indicate that the head is positioned further backward and better aligned with the upper trunk, reflecting a more neutral or posterior head posture. This or very similar measures, using the tragus of the ear as the cranial reference, are commonly used to assess head posture in ergonomic and musculoskeletal studies [36] and have shown reliable measurements [37].
• Thoracic kyphotic curvature: Measured following the methodology of Leroux et al. [35] using AutoCAD^®^ software(AutoCAD 2019, version 23.0) (Autodesk, Inc., San Francisco, CA, USA). A line was drawn connecting T2 and T12, and a perpendicular line was extended from the apex of the curve (the most posterior spinous process) to this line. The perpendicular line divided the posterior curve into two angles, whose sum represented the thoracic kyphotic angle. Increasing angular values correspond to an increased thoracic kyphotic curvature. When compared with radiographic assessment—the gold standard—the intraclass correlation coefficient obtained with this method was 0.94. The mean absolute difference was 5° (SD 4°) [35].
• Lumbar lordotic curvature: Measured using the same method as for thoracic kyphotic curvature, but considering the markers placed at T9 and S1 [35]. Increasing angular values correspond to an increased lumbar lordotic curvature. When compared with radiographic assessment—the gold standard—the intraclass correlation coefficient obtained with this method was 0.91. The mean absolute difference was 6° (SD 6°) [35].
• Horizontal pelvic alignment: Defined as the angle formed between the line connecting the anterior superior iliac spine and the posterior superior iliac spine, and a horizontal reference line. This measure is used to assess whether the pelvis is in anterior tilt (anteversion) or posterior tilt (retroversion) [36] and it has been considered a reliable measurement [38]. Increasing angular values correspond to a greater anterior tilt of the pelvis.

We recorded exposure to awkward postures associated with each job position, determining each participant’s exposure to this specific risk in accordance with the specifications of the Interterritorial Council of the National Health System in the Protocol for specific health surveillance of workers exposed to awkward postures [39]. In this protocol, exposure to awkward postures is assessed based on the presence or absence of the following criteria: fixed or restricted body positions that place excessive strain on muscles and tendons; positions that load the joints asymmetrically; and positions that generate static loads on the muscles. If a participant met any of these criteria, they were considered exposed to awkward postures [39].

### 2.3. Statistical Analysis

For the descriptive analysis of the quantitative variables, indices such as the means and standard deviations were used. For the qualitative variables, a frequency analysis was conducted to determine percentages.

Univariable general linear models were constructed to model sagittal posture parameters of the spine as a function of the exposure to awkward postures. In each model, age, job seniority, body mass index and the intensity of physical activity were included as potential confounders. Eta squared (η^2^) was used as measure of the effect size in the models. It was interpreted categorized as: η^2^ < 0.01: “negligible”; 0.01 ≤ η^2^ < 0.06: “small”; 0.06 ≤ η^2^ < 0.14: “medium”; η^2^ ≥ 0.14: “large” [40].

Statistical significance was established at *p* < 0.05. SPSS version 25.0 for Mac (IBM Corporation, Armonk, NY, USA) was used for the calculations. R version 4.5.2 (R Foundation for Statistical Computing, Vienna, Austria) was used to create the forest plot of regression coefficients (B) with 95% confidence intervals.

## 3. Results

The total number of participants in the study was 116 subjects (Table 1). There were no dropouts, and all tests were performed. The mean age was 45.86 (±5.68) years (Table 1). The length of service in the company (seniority) was 20.94 (±5.75) years (Table 1). The mean BMI of the sample was 27.15 (±3.57) (Table 1). The mean intensity of the physical activity was 12,106.53 (±11,664.95) MET-minutes per week (Table 1). Regarding exposure to awkward postures 84.50% of the sample was exposed (Table 1).

The general linear model parameter estimates analyzing horizontal head alignment as a function of exposure to awkward postures are given in Table 2. We found no statistical significance for the potential confounders: age, seniority, and physical activity. The BMI showed a negative significant relationship with the horizontal head alignment (*p* = 0.001), demonstrating a medium effect size (η^2^ = 0.090). A decrease in BMI (β = −0.517) was associated with an increase in the angle defining horizontal head position. Exposure to awkward postures was not a significant predictor of horizontal head alignment (*p* = 0.311). The model assumptions were met. Levene’s test of equality of error variances indicated that the assumption of homogeneity of variances was met (*p* = 0.767). The modified Breusch–Pagan test for heteroscedasticity proved the null hypothesis that the variance of the errors did not depend on the values of the independent variables (*p* = 0.642).

**Figure 3:**
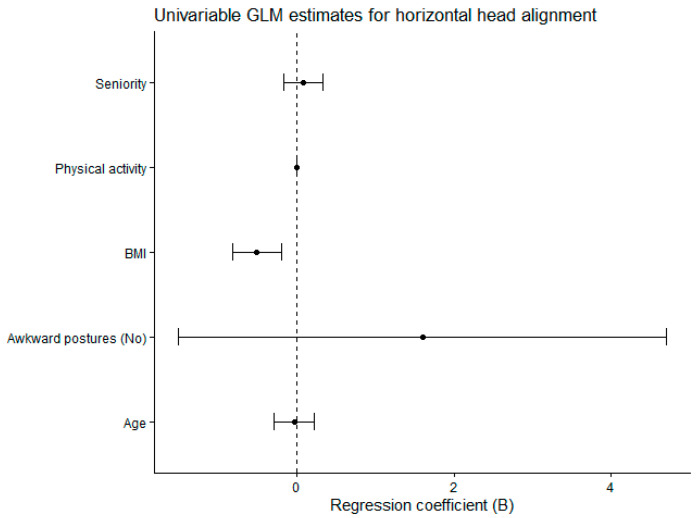
Forest plot of regression coefficients (B) with 95% confidence intervals derived from the univariable general linear model of horizontal head alignment. GLM: General Linear Model. BMI: Body Mass Index.

Figure 3 shows the forest plot of regression coefficients (B) with 95% confidence intervals derived from the univariable general linear model of horizontal head alignment.

The general linear model parameter estimates analyzing thoracic kyphotic curvature as a function of exposure to awkward postures are given in Table 3. We found no statistical significance for the potential confounders: age, seniority, BMI and physical activity. For the variable representing exposure to awkward postures (Awkward postures = No, not exposed; Awkward postures = Yes, exposed. Reference category = Yes), the regression coefficient was β = −6.479. This indicates that participants not exposed to awkward postures had, on average, 6.479 units lower in the thoracic kyphotic curvature angle compared with those exposed (reference category), after controlling for the other variables in the model. The effect reached marginal statistical significance (*p* = 0.050). The 95% confidence interval (−12.966 to 0.009) included zero at the upper bound, suggesting weak evidence but indicating a potential effect of non-exposure compared with exposure. Exposure to awkward postures, that is, the reference category (exposed), was associated with higher values in the measurement of the thoracic kyphotic curvature angle. Given the clinical interpretation of this measurement, in which greater angular values correspond to augmented thoracic kyphotic curvature, participants exposed to awkward postures presented with a higher kyphotic curvature. The effect size was moderate (η^2^ = 0.034), indicating that exposure accounted for approximately 3.4% of the variance in the dependent variable, after adjusting for other covariates. These findings suggest that, within this model, exposure to awkward postures was the variable exerting the greatest influence on thoracic kyphotic curvature, although with a moderate effect and marginal statistical significance. The model assumptions were met. Levene’s test of equality of error variances indicated that the assumption of homogeneity of variances was met (*p* = 0.302). The modified Breusch–Pagan test for heteroscedasticity proved the null hypothesis that the variance of the errors did not depend on the values of the independent variables (*p* = 0.170).

**Figure 4:**
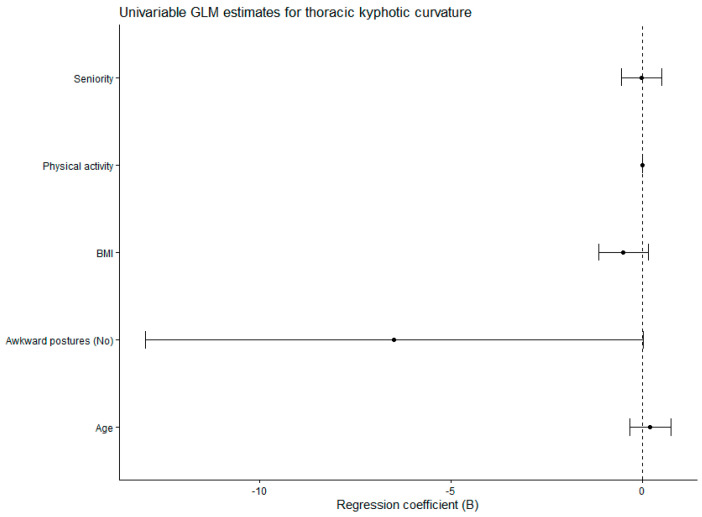
Forest plot of regression coefficients (B) with 95% confidence intervals derived from the univariable general linear model of thoracic kyphotic curvature. GLM: General Linear Model. BMI: Body Mass Index.

Figure 4 shows the forest plot of regression coefficients (B) with 95% confidence intervals derived from the univariable general linear model of thoracic kyphotic curvature.

The general linear model parameter estimates analyzing lumbar lordotic curvature as a function of exposure to awkward postures are given in Table 4. We found no statistical significance for the potential confounders: age, seniority, BMI and physical activity. Exposure to awkward postures was not a significant predictor of lumbar lordotic curvature (*p* = 0.739). The model assumptions were met. Levene’s test of equality of error variances indicated that the assumption of homogeneity of variances was met (*p* = 0.286). The modified Breusch–Pagan test for heteroscedasticity proved the null hypothesis that the variance of the errors did not depend on the values of the independent variables (*p* = 0.322).

**Figure 5:**
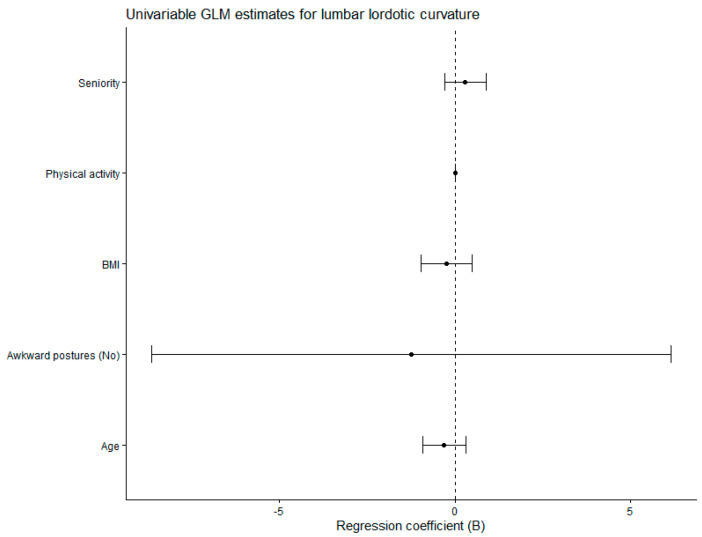
Forest plot of regression coefficients (B) with 95% confidence intervals derived from the univariable general linear model of lumbar lordotic curvature. GLM: General Linear Model. BMI: Body Mass Index.

Figure 5 shows the forest plot of regression coefficients (B) with 95% confidence intervals derived from the univariable general linear model of lumbar lordotic curvature.

The general linear model parameter estimates analyzing horizontal pelvic alignment as a function of exposure to awkward postures are given in Table 5. We found no statistical significance for the potential confounders: age, seniority, BMI and physical activity. Exposure to awkward postures was not a significant predictor of horizontal pelvic alignment (*p* = 0.220). The model assumptions were met. Levene’s test of equality of error variances indicated that the assumption of homogeneity of variances was met (*p* = 0.639). The modified Breusch–Pagan test for heteroscedasticity proved the null hypothesis that the variance of the errors did not depend on the values of the independent variables (*p* = 0.346).

**Figure 6:**
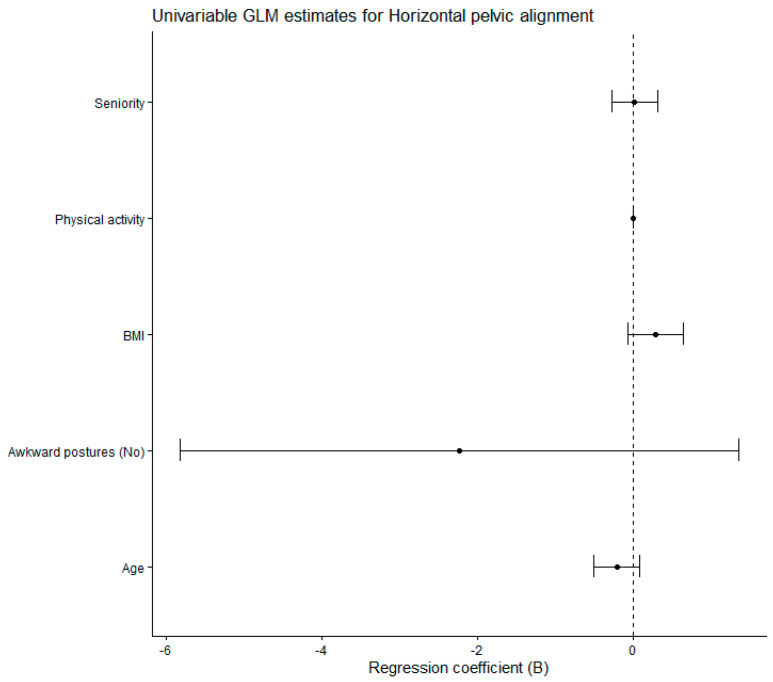
Forest plot of regression coefficients (B) with 95% confidence intervals derived from the univariable general linear model of horizontal pelvic alignment. GLM: General Linear Model. BMI: Body Mass Index.

Figure 6 shows the forest plot of regression coefficients (B) with 95% confidence intervals derived from the univariable general linear model of horizontal pelvic alignment.

## 4. Discussion

The demographic characteristics of the sample indicate that, with a mean age of 45.86 (±5.68) years, the study population is representative of a typical workforce in a Eurozone country. In the European context, the average age of workers is projected to reach 42.6 years by 2030 [41]. In Spain, data from the 2021 European Working Conditions Survey show that 55.2% of workers fall within the 31- and 50-year age range [42], and that 50.1% of employed individuals are older 45 years [43].

Our findings suggest that, in adult men employed in the industrial manufacturing sector, reductions in BMI are associated with increased angles of horizontal head position. This relationship indicates that lower BMI may favor a head position that is more posterior and better aligned with the upper trunk, while higher BMI may contribute to a more anterior head posture. Similar associations have been reported by Shaghayeghfard et al. [44], who observed a reduction in the craniocervical angle—defined using anatomical references and spatial coordinates equivalent to those of horizontal head alignment- among individuals with higher BMI values [44,45,46]. Several mechanisms may explain this association. Increased cervical and thoracic flexion has been documented in overweight individuals [47], potentially resulting from the additional mechanical load associated with higher body mass or from deconditioning of the spinal extensor musculature, both of which may hinder the maintenance of upright posture [47]. Furthermore, increased use of electronic devices has been linked to greater neck flexion [48]. Molaeifar et al. [46] suggested that weight gain leads to a proportional increase in head mass, thereby increasing cervical torque when the head shifts anteriorly relative to the vertical axis. Increased thickness of the subcutaneous fat layer in individuals with higher BMI has also been proposed as a contributing factor. Although care was taken to place the C7 marker on a clearly identifiable anatomical landmark, forward head posture in participants with higher BMI may be slightly exaggerated due to soft tissue thickness, which should be considered when interpreting these results. Additionally, compensatory scoliotic, kyphotic, or lordotic adaptations observed in obese individuals may promote cervical anteversion [49]. The influence of BMI on the stiffness and elasticity of superficial neck muscles has also been highlighted by Kocur et al. [50], who further identified age as an additional factor associated with reductions in the craniocervical angle.

It is important to note that several of the aforementioned studies were conducted in seated positions, whereas the present study assessed posture in a standing position. Sitting has been shown to decrease the craniocervical angle, thereby promoting a more forward head posture [51]. Occupational variability within the sample may also have influenced head posture, as certain tasks required sustained visual attention and concentration (e.g., fastening mattresses or operating forklifts), whereas others did not (e.g., pushing carts or transporting materials).

These findings point to a possible association between body composition and cervical posture, suggesting that body weight could play a role among other factors in ergonomic and occupational health considerations.

Exposure to awkward postures was associated with a modest increase in thoracic kyphotic curvature. Although the 6.48° difference between exposed and non-exposed workers was of borderline statistical significance, its small magnitude suggests limited clinical relevance, while still reflecting subtle postural adaptations related to occupational constraints. Moreover, the mean thoracic kyphotic curvature in the sample (42.52 ± 12.35°) was within the normal range for thoracic kyphotic curvature (20–50°) [52]. The increase in thoracic kyphotic curvature associated with awkward postures observed in this study may be explained by several factors, including work with arm advancement (for instance, work at certain stations where parts of the mattress, such as the covers, need to be moved; or handling materials on shelves), or standing for long periods of time which has been shown to increase thoracic kyphotic curvature [51,52]. Lumbar compensatory mechanisms may also play a role [53], although random variation cannot be excluded [54]. Further research incorporating additional covariates is warranted to clarify these associations. The literature addressing the relationship between awkward postures and variations in thoracic kyphotic curvature remains limited. Ohlendorf et al. [55,56,57,58] investigated postural adaptations across various professions, predominantly among dentists, who exhibit elevated risks of musculoskeletal disorders due to sustained forward-flexed postures.

Thoracic kyphotic curvature has been previously studied, with no clear differences between genders [59,60,61]. Age does appear to be a determining factor in the evolution of the angle of thoracic kyphotic curvature, especially after the age of 60 [62,63]. This age-related increase is characterized by changes in soft tissues and changes in bone mineralization over the years [64,65]. In our study, the population had a maximum age of 61 years, so this increase in kyphosis may not be related to age. BMI has commonly been referred to as one of the causes of increased kyphosis [66,67]. Increased body mass imposes greater axial loading on the spine, promoting forward trunk inclination, increased thoracic kyphotic curvature, anterior displacement of the center of gravity, and greater head advancement; however, this association was not observed in the current study [12,49].

Our study found no association between thoracic kyphotic curvature and the physical activity performed by workers. The Framingham study also found no association between physical function and kyphosis in men either older or younger than 65 years of age [68].

It is also important to note that thoracic kyphotic curvature is closely related to body posture. Previous studies have shown that sitting and lying down positions are associated with reduced kyphotic angles, whereas the standing position itself leads to an increase in thoracic kyphotic curvature in the same individuals [69,70]. Therefore, postural variation and frequent changes in body position may represent a simple preventive strategy.

In the present study, neither lumbar lordotic curvature nor horizontal pelvic alignment was significantly associated with awkward postures. Most existing research on lumbar lordotic curvature in relation to awkward postures has focused on seated work [71,72,73] limiting direct comparison with the predominantly standing tasks assessed here. Whistance et al. [74] reported postural adaptations in bipedal workers characterized by reductions in lumbar lordotic curvature, while Ghasemi et al. [75] emphasized the role of lower limb positioning in such adaptations.

Awkward postures, as defined in the Spanish occupational health framework [39], were identified in 84.5% of the sample. This prevalence exceeds the 57% reported for industrial activities in the 2015 National Survey of Working Conditions [76], indicating a higher exposure level in the present population and supporting the need for targeted interventions to mitigate this occupational risk.

Certain limitations qualify the findings of this study. Foremost is its cross-sectional nature; while a longitudinal design was deemed impractical due to anticipated sample availability challenges, this design choice inherently restricts our ability to establish causal relationships. Additionally, the study’s execution in a single geographic locale poses a challenge for generalizing results to diverse regions. Nonetheless, we think that the characterization of the study population performed allows to identify contexts where similar findings could plausibly emerge. Awkward posture exposure was categorized dichotomously (yes/no), which may have oversimplified the exposure construct. Future studies should incorporate quantitative exposure metrics, such as time-weighted duration and frequency of awkward postures, ideally using objective measurement techniques (e.g., inertial sensors or video-based posture analysis). Previous occupational exposures prior to employment in the current company were not assessed. Although seniority was used as an indicator of cumulative exposure, lifetime occupational exposure history was not available. Therefore, potential cumulative biomechanical load from previous jobs could not be accounted for. Future longitudinal studies should include detailed work history to better characterize cumulative exposure. Postural parameters were assessed using photogrammetric measurements with external skin markers. Although this method has been shown to be reliable, soft tissue artefacts and marker placement errors, particularly in individuals with higher body mass, may have introduced measurement bias.

## 5. Conclusions

Our findings suggest that, in adult men employed in the industrial manufacturing sector, lower BMI may favor a head position that is more posterior and better aligned with the upper trunk. These results underscore the potential biomechanical interplay between body composition and cervical posture, highlighting the importance of considering weight management as part of ergonomic and occupational health strategies. Additionally, exposure to awkward postures was associated with a modest increase in kyphotic curvature, suggesting limited postural adaptations in response to occupational constraints. This association was demonstrated to be independent of age, job seniority, BMI and intensity of physical activity.

## Figures and Tables

**Table 1:** Descriptive characteristics of the sample.

	Total (*n* = 116)
Awkward postures	84.50%
Age (years)	45.86 (±5.68)
Seniority (years)	20.94 (±5.75)
BMI	27.15 (±3.57)
Physical activity (MET-minutes per week)	12,106.53 (±11,664.95)
Horizontal head alignment (degrees)	36.17 (±5.96)
Thoracic kyphotic curvature (degrees)	42.52 (±12.35)
Lumbar lordotic curvature (degrees)	52.51 (±13.56)
Horizontal pelvic alignment (degrees)	11.24 (±6.73)

BMI: Body Mass Index. MET: Metabolic Equivalent of Task. Data are presented as percentages (%) or mean (±SD). The outcomes of the univariable general linear models are presented in Table 2, Table 3, Table 4 and Table 5.

**Table 2:** Univariable general linear model analyzing horizontal head alignment as a function of exposure to awkward postures. Parameter estimates.

Univariable Analysis. Dependent Variable: Horizontal head alignment	B	95% Confidence	η^2^ Effect Size	*p* Value
		Interval for B Lower Bound/Upper Bound		
Age (years)	−0.037	−0.294/0.220	0.001	0.774
Seniority (years)	0.081	−0.170/0.332	0.004	0.523
BMI	−0.517	−0.827/−0.206	0.090	0.001
Physical activity (MET-minutes per week)	6.560 × 10^−6^	−8.994 × 10^−5^/<0.001	<0.001	0.893
Awkward postures = No	1.597	−1.512/4.706	0.009	0.311
Awkward postures = Yes	Reference category			

**Table 3:** Univariable general linear model analyzing thoracic kyphotic curvature as a function of exposure to awkward postures. Parameter estimates.

Univariable Analysis. Dependent Variable: Horizontal head alignment	B	95% Confidence	η^2^ Effect Size	*p* Value
		Interval for B Lower Bound/Upper Bound		
Age (years)	0.201	−0.336/0.737	0.005	0.460
Seniority (years)	−0.021	−0.545/0.503	<0.001	0.937
BMI	−0.499	−1.147/0.148	0.021	0.129
Physical activity (MET-minutes per week)	5.256 × 10^−5^	<0.001/<0.001	0.002	0.606
Awkward postures = No	−6.479	−12.966/0.009	0.034	0.050
Awkward postures = Yes	Reference category			

**Table 4:** Univariable general linear model analyzing lumbar lordotic curvature as a function of exposure to awkward postures. Parameter estimates.

Univariable Analysis. Dependent Variable: Lumbar Lordosis	B	95% Confidence	η^2^ Effect Size	*p* Value
		Interval for B Lower Bound/Upper Bound		
Age (years)	−0.309	−0.921/0.302	0.009	0.318
Seniority (years)	0.294	−0.303/0.890	0.009	0.332
BMI	−0.243	−0.981/0.495	0.004	0.515
Physical activity (MET-minutes per week)	−7.506 × 10^−6^	<0.001/<0.001	<0.001	0.948
Awkward postures = No	−1.244	−8.636/6.147	0.001	0.739
Awkward postures = Yes	Reference category			

**Table 5:** Univariable general linear model analyzing horizontal pelvic alignment as a function of exposure to awkward postures. Parameter estimates.

Univariable Analysis. Dependent Variable: Horizontal Pelvic Alignment	B	95% Confidence	η^2^ Effect Size	*p* Value
		Interval for B Lower Bound/Upper Bound		
Age (years)	−0.211	−0.508/0.085	0.018	0.161
Seniority (years)	0.018	−0.272/0.307	<0.001	0.903
BMI	0.288	−0.070/0.646	0.023	0.114
Physical activity (MET-minutes per week)	5.299 × 10^−5^	−5.833 × 10^−5^/<0.001	0.008	0.348
Awkward postures = No	−2.232	−5.819/1.354	0.014	0.220
Awkward postures = Yes	Reference category			

## Data Availability

The original contributions presented in the study are included in the article, further inquiries can be directed to the corresponding author.

## Abbreviations

The following abbreviations are used in this manuscript:

BMI	Body Mass Index
MET	Metabolic Equivalent of Task
